# Promoting long-acting reversible contraception among post-abortion clients with a provider-comparison intervention: a cluster randomized controlled trial in Nepal

**DOI:** 10.1186/s12889-024-19150-0

**Published:** 2024-07-16

**Authors:** Jeremy Barofsky, Hannah Spring, Pragya Gartoulla, Raman Shrestha, Sabitri Sapkota, Elizabeth McElwee, Kathryn Church, Saugato Datta, Karina Livingston

**Affiliations:** 1 ideas42, Madison, WI USA; 2 ideas42, Seattle, WA USA; 3 Monash University, Southbank, Australia; 4 Sunaulo Parivar Nepal, Kathmandu, Nepal; 5 Possible Health Nepal, Executive Director, Kathmandu, Nepal; 6 ideas42, Washington, DC, USA; 7 Independent Consultant (current), Director of Global Evidence (former) MSI Reproductive Choices, London, UK; 8 ideas42 (former), Somerville, MA, USA; 9 ideas42 (former), San Francisco, CA, USA

**Keywords:** Behavioral science, Post-abortion care, Long-acting reversible contraception (LARC), Provider behavior change, Peer-comparison

## Abstract

**Background:**

Although long-acting reversible contraception (LARC) is more effective and longer lasting than short-acting methods, uptake remains low among post-abortion clients. Using a stepped-wedge, cluster-randomized trial, we evaluate the impact of a provider-level peer-comparison intervention to encourage choice of LARC in Nepal among post-abortion clients.

**Methods:**

The intervention used prominently displayed monthly posters comparing the health clinic’s previous month performance on LARC uptake against peer clinics. To understand how the intervention affected behavior, while ensuring voluntarism and informed choice, we used mystery client visits, in-depth provider interviews, and client exit survey data. The trial examined 17,680 post-abortion clients in 36 clinics in Nepal from July 2016 to January 2017. The primary outcome was the proportion of clients receiving LARCs. Statistical analysis used ordinary least squares (OLS) regression with ANCOVA estimation to assess the intervention’s impact on LARC uptake while controlling for client- and clinic-level characteristics.

**Results:**

The intervention increased LARC use among post-abortion clients by 6.6% points [95% CI: 0.85 to 12.3, p-value < 0.05], a 29.5% increase in LARC use compared to control clinics. This effect persisted after the formal experiment ended. Analysis of provider and client experiences showed that the behavioral intervention generated significant change in providers’ counseling practices, motivated the sharing of best practices. Quality of care indicators either remained stable or improved.

**Conclusion:**

We find that a provider-level behavioral intervention increases LARC uptake among post-abortion clients. This type of intervention represents a low-cost option to contribute to reducing unmet need for contraception through provider behavior change.

**Supplementary Information:**

The online version contains supplementary material available at 10.1186/s12889-024-19150-0.

## Background

Sexual and reproductive health (SRH) services, including access to modern family planning (FP) methods, are essential to the “well-being and autonomy of women” [1]. FP also positively impacts a women’s social and labor market outcomes [2]. Although the use of contraception has increased significantly in low- and middle-income countries (LMICs), over 200 million women still report an unmet need for contraception services [3]. High unmet need for FP leads to unwanted pregnancies which may end in abortion, with an average of 56 million induced abortions occurring annually, both safe and unsafe [4]. If abortion services are not available, unintended pregnancies that result in births are associated with inadequate prenatal care and childhood vaccination, as well as higher neonatal mortality [5] with 4.7% of maternal deaths linked to pregnancies with abortive outcomes (abortion or miscarriage) [6]. Unmet need remains high among some sub-populations whose needs are likely not well-served by existing SRH services, such as women who are post-abortion [4].

To meet these needs, we developed a behavioral intervention to promote contraception uptake among post-abortion women in Nepal because this subpopulation exhibits clear demand for effective contraceptives and has high levels of unmet need [7, 8]. In 2014, it was estimated that over 323,000 Nepali women received an abortion [8]. Multiple abortions remain common in Nepal, even with evidence that this may be a sub-optimal means of regulating fertility, with one study finding that one-third of abortion clients have had multiple abortions [9]. Long-acting reversible contraceptives (LARCs) are a good alternative to multiple abortions. LARCS are often under-utilized within FP programs due to a range of issues including provider training and competency, stock-outs, higher upfront user costs, perceived side effects, and/or misconceptions by clients or their partners. The important benefits of LARCs include ease of use, higher efficacy, higher cost-effectiveness, and potential for long-term use. Typical use failure rates for LARCs are less than 1% versus short term methods (STM) failure rates of up to 10% [10, 11]. These benefits of LARCs are often undervalued resulting in method skew towards STMs [12]. A study across 21 LMICs shows that rates of LARC uptake among married women range from 67% to less than 3% [13]. Nepal is a low-income country where unmet need for family planning (FP) remains high by global standards and reductions in unmet need have stagnated in recent years [14]. Uptake of LARC is low overall in south and southeast Asia. Rates for married women range from 3.1 to 11.7%, while Nepal’s rate of LARC use is among the lowest in the region at 5% [13]. In contrast, LARC use rates are 14% in West Africa and almost 50% in the Middle East and Central Asia among nations that collected recent Demographic and Health Surveys [13]. It is therefore likely that the current method mix in Nepal is suboptimal, both overall and for the specific sub-population in this study: post-abortion clients, 71% of whom take up either no contraception or STMs [7]. Among post-abortion clients across all of Nepal, 30% used LARC in June 2016, the month immediately preceding the start of our study [15].

Therefore, to counter a skew towards STMs among post-abortion clients, the primary outcome selected in this study was LARC uptake. Critically, however, all women in the study were counseled on all methods, and women could select whichever method they preferred. This was monitored with mystery client visits pre- and post-intervention, in-depth provider interviews, and annual client exit surveys.

Like many other LMICs, the legal and policy environment around contraception and FP in Nepal has become more supportive over the past few decades. Abortion was legalized in Nepal in 2002, and in the last decade substantial investments have been made in health worker recruitment and training. Nepal’s Ministry of Health has also expanded efforts to make contraceptives available at all levels of health facilities and at the community level through female community health volunteers [16]. Nevertheless, one third of clients in Nepal reported receiving no information on effective contraception methods after their abortion [17]. When providing post-abortion SRH counseling, health providers must strike a delicate balance. On the one hand, they must support decision-making by guiding clients through a complex array of contraceptive options to achieve client-specific objectives. On the other, providers are subject to structural factors such as power imbalances – e.g.: clients often defer to their judgement – or limited time and bandwidth to provide needed counseling [18]. We therefore strove to better understand the provider perspective through qualitative interviews, with a particular focus on how providers engaged with the peer-comparison intervention in this study. In addition to provider counseling, contraceptive choices vary based on a woman’s individual fertility goals, preferences, health needs, and side effects, as well as their familial and cultural environment.

The institutional setting for our study is SRH clinics run by Sunaulo Parivar Nepal (SPN), an affiliate of MSI Reproductive Choices (MSI, formerly Marie Stopes International), in 32 districts of Nepal. SPN is one of Nepal’s largest non-governmental SRH clinic networks, providing one third of total safe abortions each year in Nepal. At the time this intervention was implemented in 2017, the government of Nepal’s Family Welfare Division was working to improve access and broaden the range of family planning methods available at all levels of the health system. One way of doing this was to improve regulatory frameworks to promote public-private partnerships with organizations like SPN, to increase quality FP services [19]. Non-governmental providers play an important role in the provision of SRH services in LMICs, making it important to devise strategies to improve service delivery in this sector. SPN provides all family planning services free of charge, meaning that cost – at least in this context – cannot explain the observed low baseline uptake of LARC among post-abortion clients. Safe abortion services require clients to provide some payment, although subsidies were available for clients who were unable to pay.

### Intervention development using a behavioral science approach

To develop the intervention, we used an iterative, participatory behavioral science approach that included three stages of background research: (1) we interviewed providers and clients to understand how behavioral barriers limited FP choice post-abortion, (2) narrowed our focus to one behavioral challenge and investigated its underlying features, and (3) designed and user tested behaviorally informed solutions to alleviate the identified challenge. For a full summary of the qualitative research and co-design undertaken to develop, refine, and user test the behavioral intervention see Spring et al. (2016) [20].

From our initial interviews, we disentangled aspects of providers’ motivation. We learned that more traditional approaches—like performance bonuses and continuous training—to increase providers’ motivation to counsel women more consistently on FP methods would not have the intended impact in significantly and sustainably changing providers’ behaviors. We also noted that providers were well-trained and understood how to counsel clients on FP using balanced counseling methods [21]. Providers demonstrated high intrinsic motivation to offer quality care and compassion for their clients, wanting to not only perform well on their jobs, but also to show respectful care for clients. From this information, we identified that providers had a clear intention-action gap: having strong intentions to provide quality care and the capability to deliver quality care, but not consistently acting on this intention by failing to counsel women on FP methods.

As we unpacked the contextual influences of this intention-action gap, we found that providers were not able to accurately assess their own performance to make the connection between consistently counseling clients on FP and post-abortion family planning (PAFP) uptake. When asked about performance, several providers shared that they received feedback, but did not know if that feedback was good or bad. In some cases, providers knew that their clinic was below the global PAFP goals, but did not associate achieving this threshold with their clinic’s performance. Another insight from the interviews and observations was that role sharing and diffusion of responsibilities among providers made it possible to assume that another provider had already provided FP counseling. By making PAFP performance salient monthly, the intervention was designed to support consistent group reflection among providers and motivate coordination to ensure counseling was offered to all clients.

Given these findings, we were able to determine several design principles to guide our intervention development, we needed to: (1) ensure that any design would enhance and not limit providers’ intrinsic motivation, (2) give providers benchmarks to contextualize performance, and (3) help providers to better coordinate as a team to address service delivery gaps encountered during clients’ service journeys. These principles helped focus our intervention design on improving provider counseling performance.

We developed hypotheses based on the behavioral concepts of reference dependence and social comparison to peers, whereby individuals often assess their performance using social cues or markers. This occurs by directly observing others’ actions to determine what is socially appropriate [22]. Peer-comparison interventions have successfully improved outcomes related to condom sales [23], energy use [24], and outdoor water consumption [25].

Acknowledging providers’ deep motivation to perform well based on their responsibilities, we aimed to highlight providers’ performance without assigning injunctive prompts. By showing where a clinic stands relative to others without assigning value judgments, we hoped our design would capitalize on the intrinsic motivation to do well (and better than others) without crowding out good intentions. This feature of our design was inspired by behavioral research demonstrating that nonmonetary incentives can better motivate job performance compared to monetary ones, particularly if the job is pro-social [26]. These social interventions to promote changes to provider behavior in the medical system have also been impactfully used with providers reducing the number of unnecessary prescriptions [27] and promoting HIV prevention [23]. By November 2015, three prototypes of a behavioral intervention to communicate performance feedback to providers were user tested and validated. Based on feedback from these user tests with eight providers, we chose the final behavioral intervention utilizing peer comparison posters for PAFP uptake.

### Intervention design

Beyond conceptual development, we designed the intervention subject to several constraints. To ensure sustainability, we focused on a low-cost, low-tech solution that was easy to update and send to clinics every month. Given scarce organizational bandwidth, we wanted to minimize the burden on providers and research staff in collecting and updating data. Lastly, the designs had to be low effort to place in the clinics, as we wanted to minimize barriers for continued use. With these constraints in mind, the final intervention took the form of an 8”x10” peer comparison poster detailing clinic performance, which was mailed to clinics in the intervention group at the beginning of each treatment month.

Based on the insight from our qualitative work that providers were both intrinsically motivated and well trained, the intervention was designed to encourage service providers to collectively reflect upon their own FP counselling behavior and practices. In particular, the posters intended to increase the salience and timeliness of a clinic’s LARC uptake performance compared to peers and start conversations among provider teams to prepare and implement action plans for more consistent counseling that respected client preferences. Upon receipt of the poster, staff were instructed to hang the poster in a central, easily accessible point in their clinic (for example, a meeting room). We also asked clinics to place a publicly visible notice to clients of the ongoing study.

**Fig. 1:**
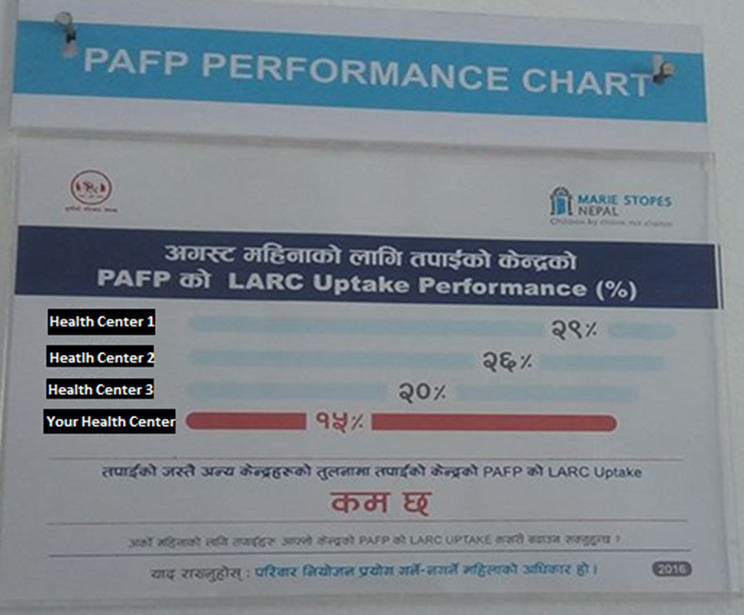
Intervention Poster in Use at an SPN clinic (Low Performing Center): 2016. **Translation -** Top section: Your Centre’s PAFP LARC Uptake Performance (%) for August. Middle section: Center 1 (names redacted for anonymity)- 29%, Center 2–26%, Center 3–20%, Your Center − 15%. Bottom section: PAFP Uptake at your Center is LOWER than that of other centers like yours. What can you do to improve your PAFP uptake for next month? Remember: It’s a woman’s right to choose whether to use family planning

**Fig. 2:**
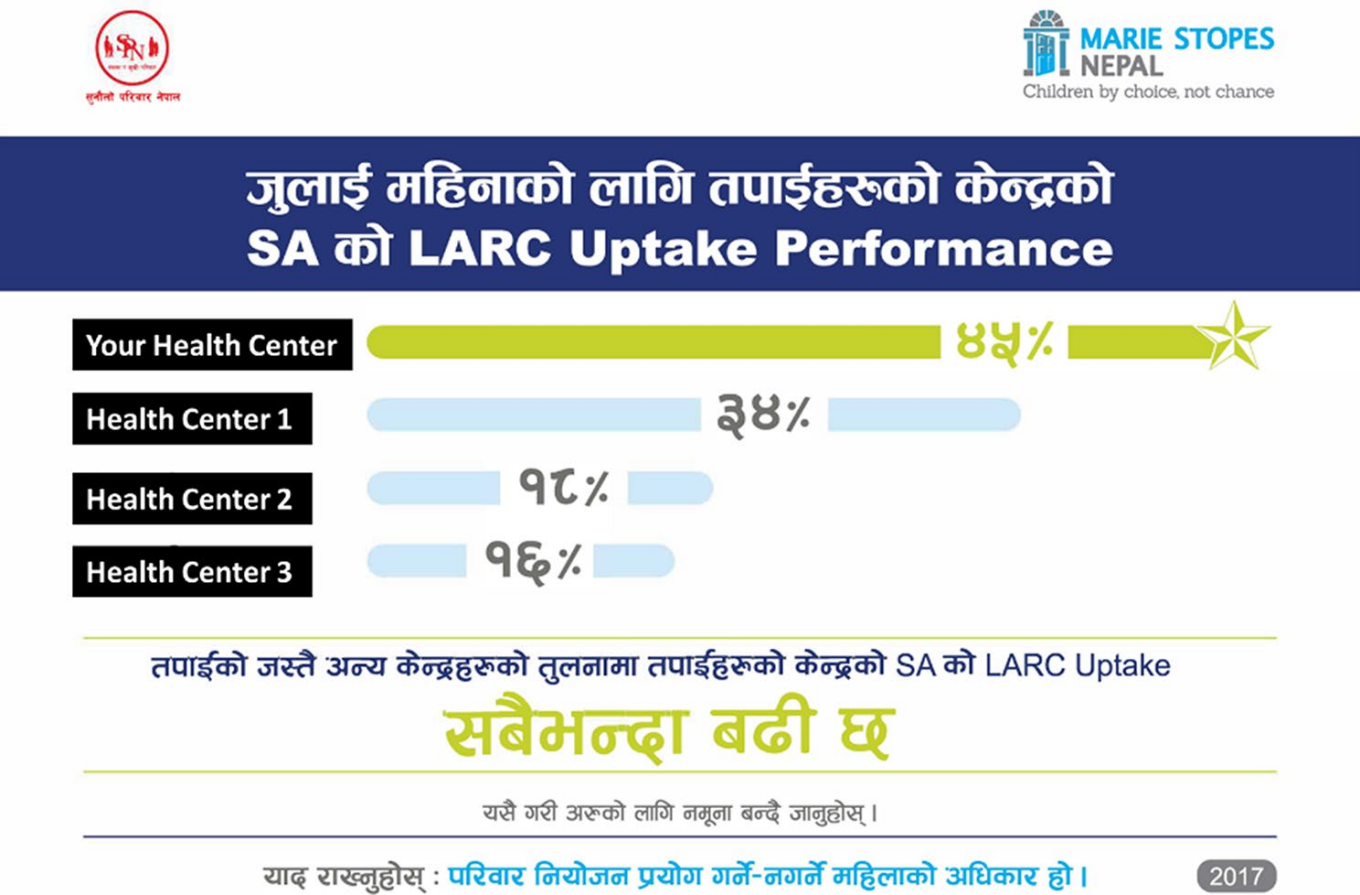
Updated Intervention Poster (example of High Performing Center): 2017. **Translation -** Top section: Your LARC Uptake for Safe Abortion (SA) in the month of July. Middle section: Your Center (names redacted for anonymity)- 45%, Center 1–34%, Center 2–18%, Center 3–16%. Bottom section: (Navy and green) Your LARC uptake for SA is HIGH compared to other similar centers. (Grey) Please continue to be an example to other centers. (Grey and blue) Please remember: To use or not use contraception is the right of the woman

The central behavioral elements of the poster were simple, visually intuitive bar graphs that compared the clinic’s post-abortion LARC uptake rates for the previous month to post-abortion LARC uptake rates in three peer SPN clinics (see Figs. 1 and 2 for poster examples in Nepali with translation). The poster was used to emphasize that LARC uptake rates approaching or reaching 100% was never plausible nor desirable in practice. This point was emphasized in the initial provider training and in monthly review meetings. In addition, all posters included the reminder: “It’s a woman’s right to choose whether to use family planning,” to preserve clients’ freedom of choice.

The monthly posters gave providers a clear benchmark to assess how their efforts translated into performance and constituted a starting point for further team discussion on how to best serve client needs. Again, this design was driven by our qualitative data collection, which indicated a desire among providers to better understand their own performance. Clinics were separated into peer comparison groups based on client flow, historical PAFP uptake, location, and number of staff to ensure that a clinic was only being compared to other clinics with similar operational profiles and characteristics. The data used in the posters were compiled from the service statistics described above. Text below the bar graph summarized the recipient clinic’s performance explicitly as either ‘high’, ‘lower’ or `lowest’. To help close the intention-action gap, we included additional text on the posters to prompt staff at low- and middle-performing centers to discuss ways to improve their post-abortion LARC uptake rate, while commending high-performing centers as role models. After the poster was posted each month, provider teams were asked to discuss the results shown on the poster and prepare action plans based on the discussion to improve performance, while always respecting client choice.

### Intervention implementation

Once the intervention design was finalized, we provided written communication to all the centers regarding the purpose of the study, how it would be implemented, and who to contact with any follow up questions. Then, before data collection commenced, the research team visited each clinic to provide a training workshop for all service providers implementing the intervention. In the trainings, the goals of the research were explained, informed consent from providers was obtained, providers were trained to consent clients, previous trainings were reinforced on respecting clients’ informed and voluntary choices, and providers were familiarized with the study’s timeline. In addition, the training described data collection procedures, including noting that providers may be interviewed on their experiences with the intervention. The training also allowed providers to have their questions and concerns answered by the research teams at SPN and ideas42, a behavioral design nonprofit organization.

Every month after the intervention commenced, data were compiled from a daily tracker, converted into performance measures on the intervention poster, and sent back to the clinics for team review and discussion. The research team confirmed if the poster was received and responded to any pending questions. Clinic teams were instructed to review the data on the posters, conduct small team meetings, and reflect upon their counselling procedures from the previous month. As needed, the clinics were encouraged to follow up with their SPN operations manager for any support or reach out to other centers for cross-clinic learnings. This same process repeated each month with posters containing the previous month’s performance data throughout the study period. Finally, a refresher training on PAFP was conducted with all center staff in early 2017 after experimental data collection was completed. The training was focused on educating providers on the most up-to-date Client-Centered Counselling and Informed Consent guidelines.

## Methods

### Sample

We collected data on the family planning behavior of all post-abortion clients that were seen in the 36 SPN clinics in this study between July 1, 2016, and January 31, 2017. The total sample size over this period was 17,680 clients. Data collection occurred by providers filling out daily registers for all clients, which included data on the SRH services received and client age. These daily registers by clinic were transferred to electronic form by SPN and transferred to ideas42 to be cleaned and merged with clinic-level characteristics. Given the stepped wedge experimental design, all clinics move from control to treatment over the course of the experiment, which means that treatment status is determined jointly by the clinic and date a client visited. A client is coded as being in treatment or control if the clinic visited was in treatment (i.e., receiving intervention posters) or still in the control group at the date of her visit. We collected data on 8,678 post-abortion clients that visited clinics during the control period and 9,002 post-abortion clients during the treatment period. There was a small amount of missingness (0.1%) on the main outcome of interest – type of family planning received – so that the main result estimates without individual-level controls have a sample size of 17,515. Missingness on individual-level variables of 2.2% means that once these controls are included in the main results, the sample size declines to 17,287.

### LARC use among post-abortion clients

Our primary outcome of interest is the proportion of post-abortion clients receiving LARC. LARC is defined in this paper as intrauterine devices (IUDs) and implants only. We define STM as including injectables, condoms, and pills. This decision reflects standard practice for the SPN clinic network implementing the intervention.

### Experimental design

The effectiveness of the finalized behavioral intervention on promoting uptake of LARCs among post-abortion clients was evaluated using a stepped-wedge, cluster-randomized controlled trial in SPN’s 36 SRH clinics spread across 32 of Nepal’s 75 districts. All 36 SPN clinics were assigned to one of four randomization groups, with nine clinics in each group. After two months during which all clinics were in the control group, one group of clinics at a time was randomized into treatment (i.e., began receiving peer feedback posters) in each subsequent month according to the stepped-wedge schedule (Table 1). Once a group of clinics was randomized into treatment, it remained in the treatment group (i.e., continued to receive monthly posters) for the remainder of the experiment. After all four randomization groups began receiving the poster, data was collected for two additional months. Therefore, data collection occurred for seven months from July 2016 to January 2017. A baseline period in which no clinics were randomized to treatment occurred from July to August 2016. From September to December 2016, one randomization group per month began receiving the group peer comparison intervention. By the beginning of December 2016, all clinics were receiving the intervention. At the end of January 2017, experimental data collection ended.

**Table 1:**
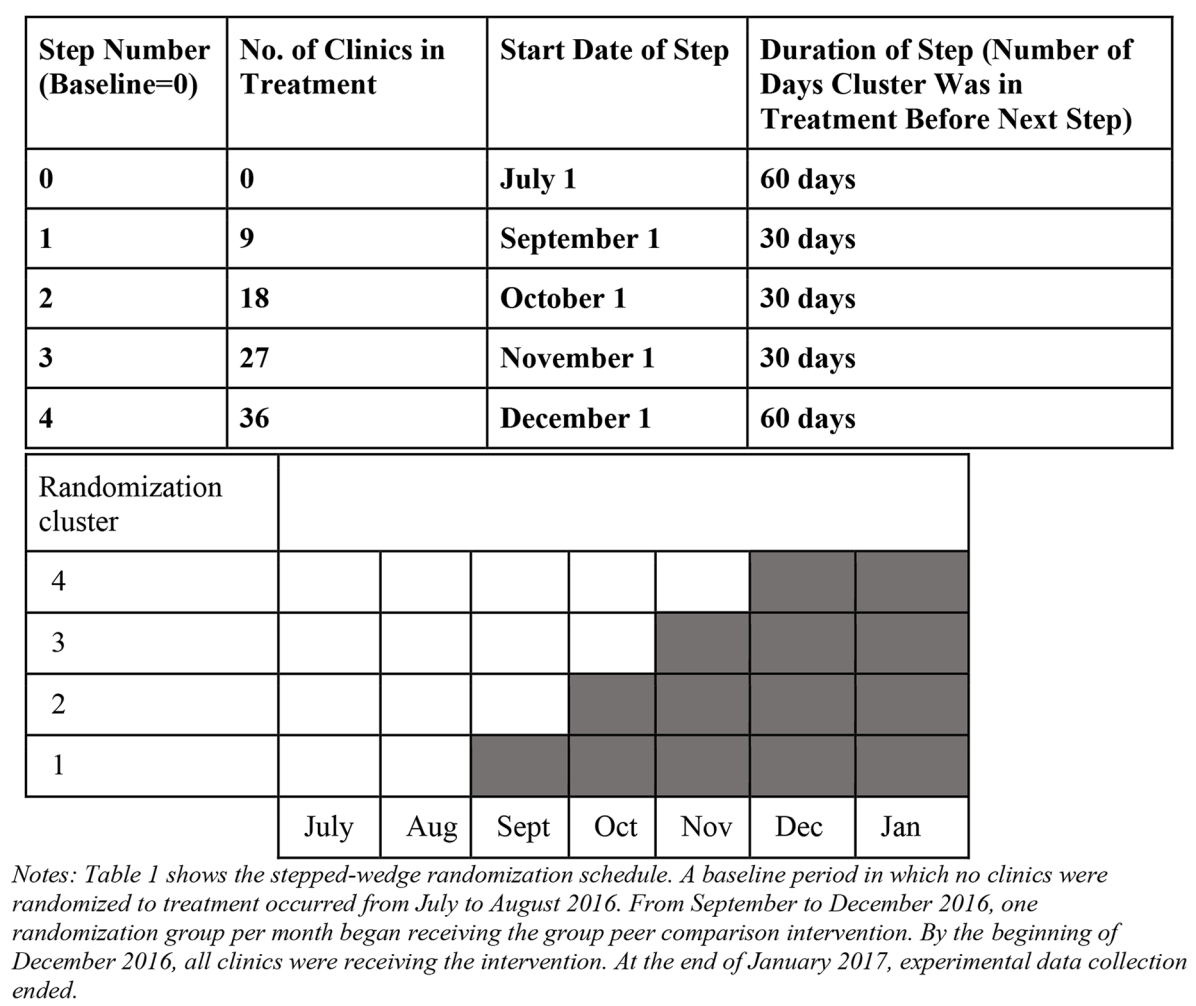
Stepped-wedge Experimental Schedule

To investigate the accuracy of the data collected in our daily tracker sheets, consent from clients was obtained to review their medical chart and research teams checked consistency in data by comparing client charts with the daily tracker sheet filled out by providers for the experiment. Approximately 20 client charts were selected in each of the 36 MSI Nepal centers over two rounds, the first during the baseline period and the second at the end of data collection. In both instances, six data fields were validated including client age, binary variables for whether the client received a medical or surgical abortion, an indicator for same-day PAFP, type of FP received, and whether the package was taken in the last 14 days.

### Analytical framework

Given that we collected panel data at the clinic level, we estimate the causal effect of the intervention on the proportion of post-abortion clients choosing LARC using ordinary least squares (OLS) regression with ANCOVA estimation [28]. ANCOVA compares the proportion of clients that receive LARC post-abortion in treatment clinics compared to control, while controlling for the baseline rate of post-abortion LARC uptake by clinic, and has been shown to improve power in randomized studies [28]. We adjust for differences in client age across clinics and time. Increasing client age is expected to be associated with greater use of LARC, but at a decreasing rate. To account for this nonlinear relationship, we include both age and age squared in the regression model. We also control for whether a client received a surgical abortion, hypothesizing that clients receiving a surgical abortion are more likely to use LARC post-abortion compared to those receiving a medical abortion. The model includes dummy variables by month and clinic to control for seasonality in LARC use and any unique clinic-specific variation in LARC uptake, respectively. A dummy variable that controls for clinics located in an urban area is also included to adjust for variation by population density. Finally, randomization group time trends are included to ensure that results are not driven by pre-existing linear trends at the randomization group level. By including month and clinic fixed effects, we identify the intervention’s effect by comparing mean LARC use in a given clinic before versus after the intervention was initiated, while also controlling for baseline LARC use and changes in client characteristics across clinics. Standard errors are clustered at the clinic level – the level of randomization – to account for within-clinic correlation in contraception use. To test model sensitivity to using OLS, we run the same model with a logistic functional form.

### Data collection to measure provider behavior and client experiences

To better understand how the intervention affected provider behavior and client care, with particular focus on monitoring clients’ informed choice, we collected and analyzed three separate data sources: (1) in-depth interviews with 18 providers across SPN clinics collected post-intervention, (2) mystery client surveys conducted pre- and post-intervention, and (3) annual client exit surveys. We describe the methods used to collect in-depth provider interviews in this paper. Methods for mystery client surveys and client exit surveys are described in the Supplement.

The in-depth provider interviews examined how providers interacted with the intervention and used it to overcome barriers to FP delivery. These interviews were also conducted to probe for any evidence that clients were pressured into specific family planning choices and to ensure that client preferences were respected. A purposive sampling approach was used to capture viewpoints from providers at clinics that differed in size, geography, and urban status. Participants were recruited from 18 of 36 treatment clinics and selected if they were providers certified to provide safe abortion services (doctors, staff nurses, and auxiliary nurse midwives). Data collection instruments were semi-structured and open-ended. The areas covered during the approximately 45-minute interviews included:

- Provider perceptions of the behavioral intervention.
- Provider behavior changes because of the intervention.
- Challenges around LARC uptake for post-abortion clients.
- Evidence for any unintended consequences from the intervention, especially whether the intervention infringed on clients’ choice.

Interviews were conducted in Nepali and recorded, transcribed, and translated into English. Transcripts were analyzed for themes and patterns and organized using Dedoose [29]. One coder, EM, developed and applied the codes using a thematic analysis approach [30]. Major themes were organized by grouping similar factor codes using the four areas above. An initial set of codes was established by the research team and in subsequent rounds of coding by EM, additional codes and sub-themes emerged inductively from the data. A second coder (AS) independently coded and reconciled the coding decisions of EM to ensure consistency.

## Ethical approval

Ethical approval was obtained from the Marie Stopes International Ethical Review Committee application ID 001–016 and Nepal Health Research Council application Reg.no. 89/2016. All research activities were performed in accordance with the protocols submitted to these institutions and in compliance with their guidelines and regulations.

## Results

### Balance by treatment status

Table 2 compares covariates between treatment and control clients for balance. We observe balance between treatment and control for individual-level variables: percent receiving a surgical abortion and age. For clinic-level variables, we find that control clients are more likely to be in urban areas and to visit clinics with slightly more staff, but these differences are not statistically significant. Because we observe balance in individual and clinic characteristics, Table 2 suggests that the randomization was effective.

**Table 2:** Means or proportions, differences in means or proportion, and 95% confidence intervals of individual and clinic characteristics while clinics were in the control versus treatment condition

	Post-abortion clients		Difference in means (95% CI)	
	Control	Treatment	Treatment - Control	
	(*N* = 8,678)	(*N* = 9,002)	(*N* = 17,680)	
	[1]	[2]	[3]	
**Surgical Abortion**	0.593	0.592	-0.0007	(-0.005–0.044)
**Age of client**	27.978	28.051	0.073	(-0.150–0.296)
**Urban**	0.700	0.625	-0.075	(-0.170–0.020)
**# of Staff per Clinic**	4.335	3.646	-0.689	(-0.310–0.172)

*Notes* Table 2 shows summary statistics for the post-abortion clients that comprise our sample (*N* = 17,680). Columns 1 and 2 show the mean of various client- and clinic-level covariates for the control and treatment sample, respectively. Column 3 shows the mean difference between treatment and control clients and its 95% confidence interval

### Data validation

Data validation revealed very low levels of discrepancies between the daily tracker sheet data used for this study and client charts. The first-round check showed the total number of discrepancies across all clinics and variables to be 56, while the second round showed a total of 38. This translates into a discrepancy rate of 1.3% in the first round and 0.88% in the second, given that, in each round, a total of 4320 data points were checked (20 clients per 36 clinics for six data fields). The discrepancies are distributed close to uniformly across clinics, with the highest clinic discrepancy rate for any clinic across the two rounds reaching 5.8%.

### LARC use among post-abortion clients

Overall, 25.5% of post-abortion clients used LARC across all months during which experimental data were collected. Without regression adjustment, 22.4% of clients received LARC in control clinics and 28.5% obtained LARC in treatment clinics. Table 3 shows the OLS regression-adjusted treatment effect for the proportion of LARC use among post-abortion clients. The peer comparison intervention increases the proportion of LARC users by 6.6 (95% CI: 0.9 to 12.3) percentage points, which represents a 29.5% increase in the proportion of post-abortion clients using LARC compared to LARC use in control clinics.

**Table 3:** Coefficients (and 95% confidence intervals) from an OLS regression assessing the effect of the provider-level intervention on LARC uptake among post-abortion clients

	Coefficients
**Treatment**	0.066 (0.009–0.123)**
	*[0.028]*
Baseline Control	0.314 (0.050–0.578) **
	*[0.130]*
Age	0.014 (0.004–0.023)***
	*[0.005]*
Age^2^	-0.001 (-0.0003 - -0.00001)***
	*[0.00008]*
Surgical Abortion	0.149 (0.118–0.181)***
	*[0.015]*
Urban	-0.237 (-0.256 - -0.218)***
	*[0.009]*
Constant	-0.300 (-0.669–0.068)
	*[0.182]*
R-squared	0.097
**N**	**17,287**

* *p* < 0.10, ** *p* < 0.05, *** *p* < 0.01

*Note Standard errors are shown in brackets below the coefficients and confidence intervals*

### Treatment effect persistence

**Fig. 3:**
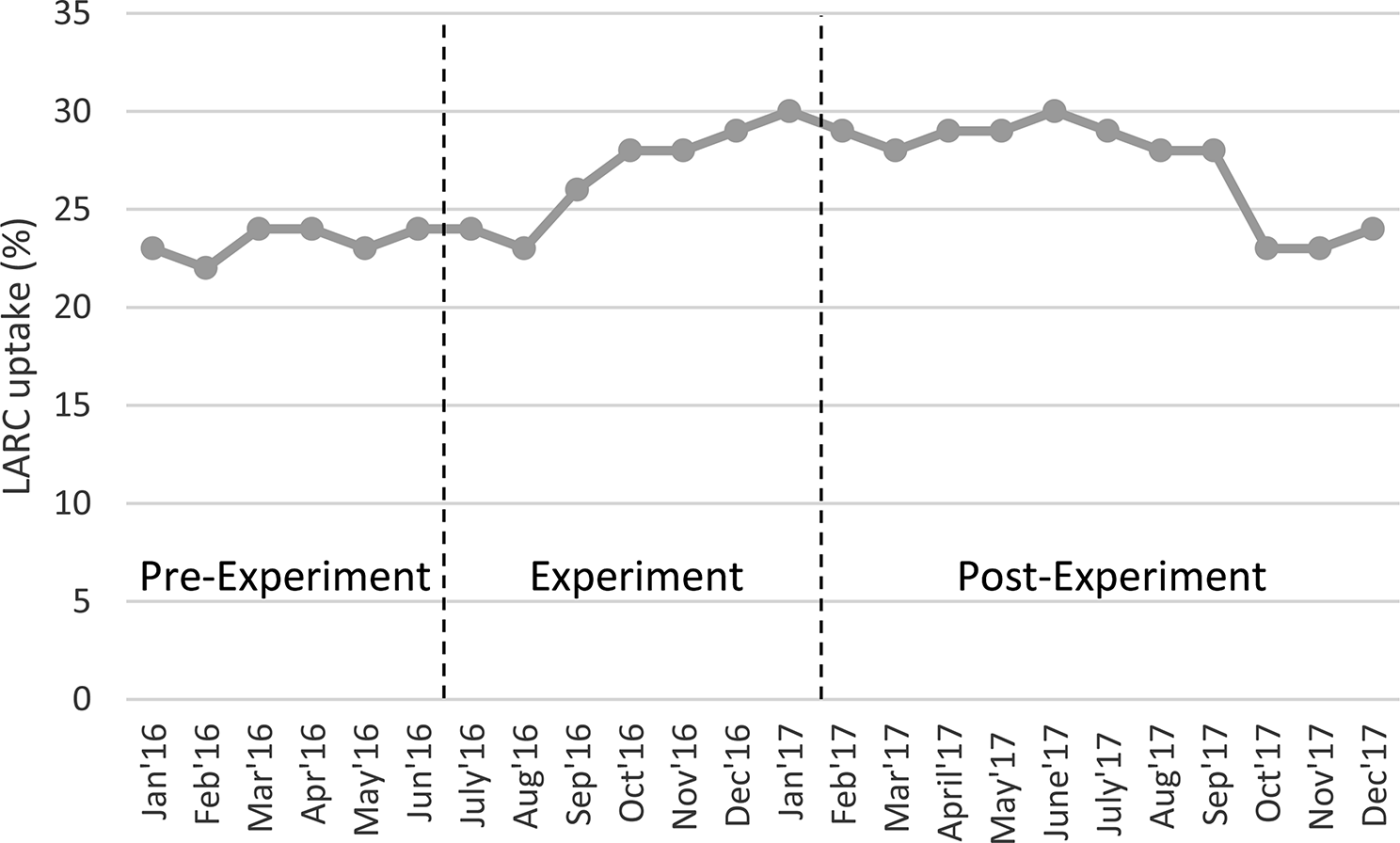
Long-term LARC uptake rate. Note Fig. 3 shows aggregate LARC uptake among post-abortion clients for study participating clinics during the pre-experimental period (January to May 2016), experimental period (July 2016 to January 2017), and post-experimental period (February 2017 to December 2017)

Further, while our client-level data collection ended in January 2017, all 36 SPN clinics in Nepal continued using the peer-comparison intervention beyond that date. After data collection for the experiment finished, clinics continued receiving peer comparison posters, but via email instead of a printed poster through the mail. Thus, practitioners still had access to the monthly peer comparison feedback posters, while programmatic monitoring continued as it had pre-intervention. Given this, we were able to investigate the persistence of the increase in LARC uptake beyond client-level data collection for the experiment. Aggregate data available until the end of 2017, summarized in Fig. 3, show that network-wide post-abortion LARC uptake rates ranged from 28 to 30%, approximately where they were by the end of the experiment, for the first seven months after the end of the experiment. They then dipped to 23% in October 2017 and remained lower until December. Given the observational nature of these data, we cannot say whether this drop reflects a reduction in the intervention’s efficacy, another change in SPN procedures, changes in client mix, or other factors. For example, the LARC use drop in October and November coincides with the period during which Nepal celebrates its principal religious festival, with associated holidays and travel to hometowns for much of the population. However, these data do indicate that LARC uptake rates generated by the behavioral intervention did not dissipate immediately after nor in the seven months after the experiment’s formal end.

### Changes in provider behavior

We interviewed 18 providers to understand how they interacted with the intervention and how this interaction affected their behavior. All interviewees reported paying attention to the information provided by the intervention’s posters. When the poster was received with the rate of post-abortion clients’ LARC uptake for the clinic from the preceding month, team members were excited to observe their performance compared to other clinics. All interviewees reported engaging with the poster, feeling motivated by it and most discussed how they could improve performance during staff meetings. The following indicates a common sentiment expressed by most interview participants:

*“First we looked at the intervention poster with curiosity. We looked together. We looked at the comparison scores. We compared it with other [clinics]. After that, all staff sat down for a meeting and we took minutes about our situation and how we need to improve it…we discussed how we can move forward.” (lowest clinic)*^1^

In addition to motivating change after the poster’s initial receipt, interviewees reported that receiving the poster sparked ongoing discussions, both within their clinics and with comparison clinics. Fifteen respondents reported increasing the frequency of their group discussions related to post-abortion family planning and LARC methods, and three facilities reported holding discussions with other centers with high LARC uptake. Ten of these began either weekly (n = 3) or monthly (n = 7) discussions. For example:

*“We see [the poster] every day since it has been hung up. Every day I think about what to do about [LARC uptake]. Daily, during our teatime, we discuss what will be fruitful for us to progress. We all feel that we need to work more on this, so we discuss how to increase [LARC uptake].” (highest clinic)*.

*“We discussed with the other clinics with high uptake on phone and asked, ‘how will you do counselling?’” (middle clinic)*.

Other respondents reported that simply knowing that LARC uptake among post-abortion clients was being measured through the poster program increased the salience of LARC uptake in their daily routine. Respondents reported that prior to receiving the poster, they were unaware of their own performance with LARC uptake among post-abortion clinics and did not even consider how their performance compared to peer clinics. Providing this performance information was useful in its own right.

*“Main message, the work that we are doing has been evaluated. We are getting the result based on our work. When I get evaluation and feedback of my work, I feel happy.” (middle clinic)*.

Additionally, respondents reported being motivated to improve based on the comparison poster. Whether they worked in clinics who on average had the lowest rates (n = 4), middle rates (n = 10) or highest rates (n = 4), every respondent expressed motivation to improve.

*“In the beginning, when we received a low position, we felt bad. But along with that we felt the motivation to do better. In December, we were in high position so that percentage also motivated us to work more. The messages written in pink motivated me even if we were low.” (lowest clinic)*.

*“We discussed about how to increase [LARC uptake], to be above other centers. We want to continue being in first position, because before we were also highest.” (highest clinic)*.

The interviews also detailed how providers changed their counseling behaviors and process. One respondent explained that after receiving the posters, the team agreed that a reason their LARC uptake performance did not increase was related to inconsistent client-centered counseling. The journey through safe abortion care includes multiple service delivery points where counseling could be provided – registration, the counseling session, post procedure, and before departure. Because of time constraints and inattention, these counseling points were not being used. Sixteen respondents reported expanding counseling to include all stages and thirteen reported focusing on balanced counselling during these sessions. An example of this can be seen in the following quotes:

*“Before, the clients used to be counselled only by the counsellor but now if any clients do not choose a method [with a counsellor], all staff give counselling from all levels.” (middle clinic)*.

*“After counselling, when [clients] come to the examination room, we do counselling there as well. We tell them about LARC methods and all family planning methods. After we provide the [abortion] service we also ask them whether they want to take any family planning devices. We again provide counselling about the importance of PAFP, especially to prevent complications of abortion.” (middle clinic)*.

In all, providers reported that the intervention prompted them to think more about LARC provision and to make sure their clients were receiving adequate counselling around PAFP and LARC. In addition to those mentioned above, several other discreet behavior changes were mentioned in the interviews as listed in Table 4.

**Table 4:** Types of behavior change mentioned in qualitative interviews with health providers

Type of behavior change	*n*
Counselling during all stages	16
Increase group discussions about PAFP and LARCs	15
Increased community outreach	3
Talk to other health centers	3
Show actual methods to clients	3
Counsel family members (husbands and mother-in-law)	2
Follow up with phone calls or give phone number	2
Giveaways to clients	2
Counsel in local language	2
Media Campaigns	2

### Challenges and unintended consequences

In the interviews, we also sought to understand the challenges to improving LARC uptake among post-abortion clients and document any unintended consequences from the intervention. The challenges identified by the providers can be separated into three categories: structural issues, clients’ attitudes towards LARC uptake, and women’s perceived lack of agency. In total, eight respondents cited structural issues in increasing LARC uptake including not having enough staff (n = 7) or not having enough time (n = 3).

*“However, because staff from this center [clinic] are sent to other centers, we cannot sit together and fully discuss our plans [for PAFP]. Hardly any staff remain in the center, they are being moved to other centers.” (middle clinic)*.

Around the issue of clients’ pre-existing attitudes and behaviors that keep clients from taking up a LARC, six respondents mentioned the following: clients do not listen either because of pain from the procedure (n = 2) or because of distrust of the health worker (n = 3); clients only trust what doctors say, not nurses or counsellors (n = 1); education level of clients affects their decisions (n = 2); and fear of side effects, either real or myths (n = 2).

*“Even if we say [PA clients] can take [LARCs], they will not agree. If they want to know one thing, they will ask all staff, not just one staff. It’s not that they don’t understand. They confirm with all staff…checking if they all say the same thing or not.” (lowest clinic)*.

Respondents also described clients’ social situation as barriers to FP use. Four respondents reported that uptake of LARC is difficult due to issues related to women’s lack of agency —either the husbands or mothers-in law disagree with FP use or they are not present at the clinic and need to be consulted before a decision is made.

*“[The women] will follow whatever their husband says [in regard to LARC use] or whatever their mother-in-law says. But when clients come, they don’t bring [their] mother-in-law, nor [their] husbands.” (highest clinic)*.

Very few respondents discussed or revealed areas in which this intervention had unintended consequences (n = 5). Of those who did, one mentioned that they feared creating divisions with the other health clinics if they discussed the posters with them:

*“It’s not good to ask [other clinics how they did on the poster] and it’s also difficult for them to tell. It might create division between staff as well. So, it’s not good to ask.” (middle clinic)*.

Four of the health providers mentioned concerns that the focus on LARC uptake may lead to provider bias in excessively promoting LARC methods, regardless of the clients’ preference. It is also possible that the health providers’ status in the community, especially the doctors, make it difficult for clients not to blindly follow a suggestion of using LARC. This was especially a worry for one provider who seemed conflicted about the focus on increasing LARC use.

*“To speak truth, it is one’s choice. We should not force if they are not willing. But since we are repeatedly telling them, it’s a bit like forcing. Isn’t it?” (middle clinic)*.

On the other hand, ten of the respondents clearly showed that they understood that even with the poster intervention focusing on LARC use, it was the client’s choice to start a LARC, use a STM, or choose no method. Even when they acknowledge that a client not taking up a LARC could decrease their rating on the poster, providers are still respecting women’s choice as the most important aspect of their work.

*“If clients do not want to take LARC, we are informing her about other methods also,” (lowest clinic).*

*“The matter of family planning should be taken on choice. It is not that we will forcefully give it to them, but they should take it based on their choice,” (middle clinic).*

*“We could not [give LARC methods] against the will of client. Due to this in some months our [position] has increased and in some months, it has decreased,” (middle clinic).*

## Discussion

This paper describes how a health provider intervention implemented through a monthly poster comparing clinic performance against peers affected uptake of long-acting reversible contraception among post-abortion clients. We find that the proportion of LARC use among post-abortion clients increased by 29.5% relative to controls. Using aggregate data, we also show that the estimated treatment effect persisted well after experimental data collection ended.

The treatment effect is clinically meaningful because it represents a substantial increase from LARC use rates compared to control clinics, while continuing to respect client preferences. To provide additional context, if we extrapolate the total number of clients to the annual level from our data (which lasted seven months) and across all MSI Nepal’s clinics (our sample includes half of those facilities), we calculate that the intervention would raise the number of post-abortion clients using LARC by 4000 annually.

To understand how the intervention affected provider behavior, we conducted qualitative interviews across 18 clinics. Systematic analysis of those interviews indicate that providers paid close attention to their LARC uptake performance compared to their peer clinics, were motivated by the information, and changed counseling behavior by offering comprehensive counselling to post-abortion clients during all points of care, from intake to discharge, to increase contraceptive choice. Throughout the design, pretesting and piloting phase of this study we were careful about potential pressure the service providers could feel that might lead to coercion compromising clients’ voluntarism and informed choice. To mitigate this, we highlighted the purpose of this intervention with the service providers and continually reinforced that they were expected to respect informed and voluntary choices as per MSI principles of service provision. This reinforcement happened during the introduction of the study, review meetings, through operations managers, and through the intervention poster itself. To identify any such coercion, we used mystery clients, client exit interviews, and in-depth providers interviews. Analyzing data from the MCs and client exit interviews, we did not find any evidence of clients’ voluntarism and informed choices being compromised, nor observe pressure felt among the service providers from the in-depth interviews.

Our findings are consistent with the literature on peer comparison interventions as a way to improve performance in other healthcare settings, which show persistent improvements in compliance to protocols in high income countries [31–33], and adds to the evidence base that this method also works in LMICs, as seen with condom sales in Uganda [23]. Previous research has found that interventions must address both societal and organizational factors together to improve the quality of family planning services [18, 34]. Although we uncovered evidence that structural or societal factors continue to impede take-up of LARC, these results indicate that when providers exhibit intrinsic motivation, a behavioral intervention can lead to better post-abortion counseling, even if structural factors remain unchanged.

The findings from this study contribute to the body of evidence around intervention strategies that increase LARC uptake by focusing on providers. While other studies focused on addressing clinical and counseling skills [35, 36] or structural factors [37], this study finds that in certain contexts, using a behavioral intervention that focuses solely on provider performance feedback with peer comparison is an effective solution to improve FP outcomes.

## Limitations

Since the data we collected did not track clients over time, our measure of post-abortion family planning captures clients who received both their abortion and contraception services at the same appointment. It is possible that our data miss post-abortion clients who came for a follow-up visit and received FP at that time. These clients would be counted not as post-abortion clients, but rather regular FP clients because there is no record of their previous abortion. Crucially, we would not expect this under-counting to vary differentially by treatment or over time and thus the omission of post-abortion women who received FP on a subsequent visit would not bias our estimate of the treatment effect. In fact, if the treatment effect among clients that follow-up in later visits was like the intervention’s effect on same-day clients, the results we report would underestimate the intervention’s overall impact.

## Conclusion

This study finds that a provider-level peer comparison intervention significantly increased LARC uptake among post-abortion women in a low-resource environment. Additional qualitative and survey data indicates that the intervention led to greater emphasis on counseling clients consistent with their goals post-abortion. Our mixed methods study supports the argument in Ashton et al. (2015) that behaviorally informed interventions can play an important role to improve family planning decision-making [38]. The intervention is of programmatic relevance because it can be readily applied in low-resource settings with access to routine data on PAFP.

The continued use of the intervention by the health delivery organization without ongoing supervision or assistance from the research team constitutes an encouraging example of both an intervention being scaled up nationally (in this case across the entire network of SPN clinics in Nepal) as well as of successful building of internal capacity within the health organization to deploy behaviorally informed interventions. At the individual level, the results presented here indicate the power of low-cost provider-focused behavioral interventions to improve post-abortion family planning using peer comparison performance feedback.

### Electronic supplementary material

Below is the link to the electronic supplementary material.

Supplementary Material 1

## Data Availability

The data that support the findings of this study are available on request from the corresponding author. The data are not publicly available due to privacy and ethical restrictions. The study was preregistered at ClinicalTrials.gov under identifier NCT03071029.

## Footnotes

1 Quotes from each clinic are represented by their average feedback position on the monthly comparison poster: highest, middle or lowest clinic.

## Abbreviations

CEIs: Client exit interviews
PAFP: Cost-abortion family planning
FP: Family planning
IUDs: Intrauterine devices
LARCs: Long-acting reversible contraceptives
LMICs: Low- and middle-income countries
MSI: MSI Reproductive Choices, formerly Marie Stopes International
MCs: Mystery clients
OLS: Ordinary least squares
SRH: Sexual and reproductive health
STM: Short term methods
SPN: Sunaulo Parivar Nepal
