# Supplementary material for: Promoting long-acting reversible contraception among post-abortion clients with a provider-comparison intervention: a cluster randomized controlled trial in Nepal
Source: BMC Public Health. 2024 Jul 16;24:1903. doi: 10.1186/s12889-024-19150-0 (PMC11251304; doi:10.1186/s12889-024-19150-0)
Supplement: Supplementary file 1 — Supplementary Material 1 [file 12889_2024_19150_MOESM1_ESM.docx]

Supplementary Materials to “**Promoting Long-Acting Reversible Contraception among Post-abortion Clients with a Provider-Comparison Intervention: A Cluster Randomized Controlled Trial in Nepal”**

1. Data Collection to Measure Provider Behavior and Client Experiences

In addition to in-depth provider interviews, we conducted mystery client surveys pre- and post-intervention to examine intervention implementation and identify any potential coercion. Mystery clients (MCs), also called standardized patients, are widely recognized as the gold standard to measure provider practice and care quality (1). MCs were trained to present to clinics with a uniform set of FP needs so that clinic responses could be consistently compared (see Exhibit S4 for a description of the three standardized scenarios used). MCs were recruited across Nepal’s geographic regions (East, Central, West, Mid-West, and Far-West) such that they were all local residents that spoke the relevant regional language. Before visiting clinics, MCs were invited to Kathmandu to attend a 3-day training where they learned how to enact the various scenarios with mock sessions and role playing, what to focus on during the observation, and how to fill out the observation checklist. Research assistants recruited MCs and verified reported responses. The MCs reported on the following provider characteristics: 1) whether the provider’s attitude was positive, 2) whether the mix of methods offered was sufficient, 3) display and explanation of methods given, 4) if freedom of choice was encouraged and, 5) if clients were given the opportunity to ask questions. That is, each MC assessed whether providers satisfied an appropriate level for each question, as defined by their training and each variable was rated as binary (yes or no). The MC surveys were conducted from July to August 2016 (pre-intervention) and again from December 2016 to January 2017 (post-intervention). Two-proportion Z tests were performed to determine whether the proportion of MCs answering in the affirmative across questions differed in the pre- versus post-period.

We analyzed client exit interviews (CEIs) to measure care quality and ensure informed choice. These interviews are collected annually by SPN and provide additional evidence on client experiences over time. Clients were randomly sampled for the exit interviews to be representative of the overall SA client population during the data collection period. The CEIs were conducted across all 36 study clinics in 2015 (pre-intervention), 2016 (when most clinics had been treated), and 2017 (one-year post-intervention) in November or December of each year. Since the intervention continued through 2017, this survey round measures the intervention’s implementation after all clinics used the intervention and had become accustomed to it. Respondents answered the survey anonymously and were assured that their responses could not be connected to their medical record. Among those for whom consent was obtained, we analyze responses to six questions that measured care quality and the content of the counseling received. Specifically, respondents were asked 1) whether they received FP counseling during their visit, 2) whether their provider explained potential side effects of the FP service received, 3) whether clear follow up instructions were given if clients experienced side effects post visit, 4) overall, how satisfied the client was with the services provided, 5) how likely was the client to recommend the facility to a friend, and 6) did the client receive the FP method they had in mind when they came to the facility (for those that had any initial FP method in mind). All questions were coded to be binary indicators of care quality (see Exhibit S5 for the full CEI questionnaire). Once transformed, we analyzed whether the proportion of responses coded to “yes” differed across survey years. Two-proportion Z tests were used as above to compare whether responses differed statistically between 2015 versus 2016 and 2015 versus 2017.

1. Logistic Regression Results

The logistic regression results on the proportion of clients using LARC can be found in Exhibit S1 below. We observe that the results that are similar in direction, magnitude, and precision to those found using OLS. Specifically, we find an odds ratio of 1.51 (95% CI: 1.13 to 2.04) for LARC uptake in treatment compared to control clinics. This represents a 51% increase in the odds of LARC usage in treatment compared to control or a 34% increase in the probability of LARC uptake from treatment, quite close to the results in Table 3 of the main document.

**Exhibit S1:** **Odds ratios (and 95% confidence intervals) from a logistic regression assessing the effect of the provider-level intervention on LARC uptake among post-abortion clients.**

|  | Odds ratio |
| --- | --- |
| **Treatment** | 1.51 (1.13 - 2.04)*** |
| *Standard Error* | *0.229* |
| Baseline Control | 9.72 (2.25 – 42.03)*** |
| Standard Error | *7.262* |
| Age | 1.10 (1.04 - 1.15)*** |
| *Standard Error* | *0.028* |
| Age^2^ | 1.00 (0.99 - 1.00)*** |
| *Standard Error* | *0.0004* |
| Surgical Abortion | 2.46 (2.08 - 2.91)*** |
| *Standard Error* | *0.2096* |
| Urban | 2.17 (0.75 - 6.29) |
| *Standard Error* | *1.177* |
| **N** | **17287** |

* p<0.10, ** p<0.05, *** p<0.01.

1. Client Experiences Results

Exhibit S2 compares findings from the clinic visits of 104 and 57 mystery clients pre- and post-intervention, respectively. All metrics of provider-client interactions measured by the MCs showed quality improvements post-intervention, and in all except one measure (opportunity to ask questions) those increases are statistically significant. Of note, the MCs found large increases post-intervention in whether providers offered a mix of methods (from 58.7% to 78.9%, p-value < 0.001), display and explanation of multiple methods (59.6% to 75.4%, p-value = 0.001), and supported freedom of choice (67.3 to 77.2%, p-value = 0.032). These results complement our findings from provider interviews that one way in which the intervention affected method choice was by encouraging providers to improve counselling quality, offer more methods with improved explanations, and encourage a broader feeling of choice.

**Exhibit S2: Results of a Z-test Means Comparison of Mystery Client Surveys**

| **Indicator** | **% of respondents satisfied** | | **p-value (z-test)** |
| --- | --- | --- | --- |
|  | **Pre- intervention (N=104)** | **Post-intervention (N=57)** |  |
| Attitude of service provider | 88.5 | 98.2 | 0.029** |
| Mix of methods offered | 58.7 | 78.9 | <0.001*** |
| Display and explanation of methods given | 59.6 | 75.4 | 0.001*** |
| Freedom of choice encouraged | 67.3 | 77.2 | 0.032** |
| Opportunity to ask questions | 89.4 | 94.7 | 0.252 |

* p<0.10, ** p<0.05, *** p<0.01.

Exhibit S3 summarizes client CEI results from 2015, 2016, and 2017. Approximately the same number of clients were sampled annually: 244, 223, and 236, respectively. We observe very high levels of quality reported during the pre-intervention period for five of six indicators. The baseline levels of quality are high enough that for most indicators ceiling effects apply, meaning that percentages could only remain the same or worsen statistically, given our sample sizes. We find that for four questions (received FP counselling, informed about side effects, received follow-up instructions, and recommend to a friend), responses immediately and one-year post-intervention cannot be distinguished statistically from their (high) pre-intervention levels using the 0.05 p-value threshold. For reported satisfaction with services overall, we find a statistically significant decline in the proportion responding yes immediately post-intervention. However, this proportion recovers to a slightly higher level than pre-intervention one-year post-intervention. Similarly, we observe a statistically significant decline immediately post-intervention in the proportion of clients that received the FP method that they had in mind when they started their visit (among clients that had any method in mind). However, given the small sample sizes (82 respondents in 2015 and 71 in 2016 and 2017) for this question, those with a specific method in mind are unlikely to be representative of the overall SA client population each year. Second, the proportion receiving their preferred method one-year post-intervention rebounds to be larger than pre-intervention percentage (86% vs 78%), although these are not statistically different. Taken together, these results on client experiences again indicate high and sustained levels of care quality was provided. They also indicate that one mechanism by which the intervention improved LARC uptake occurred through better counselling quality.

**Exhibit S3: Results of Z-test Mean Comparisons of Client Exit Interview Responses**

| **CEI** | **Percentage (95% C.I.)** | | | |  |
| --- | --- | --- | --- | --- | --- |
|  | **2015**  **(N=244)** | **2016**  **(N=223 )** | **p-value**  **(2015 vs 2016)** | **2017 (N=236)** | **p-value**  **(2015 vs 2017)** |
| Received counselling on FP during visit | **96%**  (92.6%, 98%) | **95%**  (90.8%, 97.2%) | 0.506 | **98%**  (95.7%,99.5%) | 0.118 |
| Informed about potential side effects | **96%**  (92.5%, 98.5%)^a^ | **92%**  (88.1%, 95.5%) | 0.903 | **99%**  (97.0%, 99.9%) | 0.041** |
| Received follow up instructions | **98%**  (94.7%, 99.1%) | **95%**  (90.1%, 97.2%) | 0.101 | **99%**  (97%, 99.9%) | 0.168 |
| Reported satisfaction with services provided | **93%**  (89.1%, 95.9%) | **84%**  (78.9%, 88.8%) | 0.003*** | **94%**  (90.2%, 96.7%) | 0.645 |
| Would recommend experience to a friend | **100%**  (98.5, 100%)^b^ | **99%**  (96.1%, 99.7%) | 0.069* | **99%**  (97.0%, 99.9%) | 0.15 |
| Received FP method that clinic had in mind at start of visit | **78%**  (67.5%, 86.4%)^c^ | **59%**  (46.8%, 70.7%)^d^ | 0.012** | **86%**  (75.6%, 93%)^d^ | 0.209 |

*Note: Client exit interviews were randomly sampled in November to December each year to represent safe abortion clients visiting all SPN clinics. ^a^ n=188, ^b^ one-sided, 97.5% confidence interval, ^c^ n=82, ^d^ n=71.*

* p<0.10, ** p<0.05, *** p<0.01.

REFERENCES:

1. Miller R, Das J, Pai M. Quality of tuberculosis care by Indian pharmacies: Mystery clients offer new insights. J Clin Tuberc Mycobact Dis. 2018 Jan 1;10:6–8.

**Exhibit S4: Standardized scenarios used for mystery client observations and questionnaire**

Scenario 1

- Personal Characteristics: Unmarried, around 22 years, college student (bachelor level), with an unplanned pregnancy
- Reason for visit: coming to the facility to ask about medical abortion (MA) to terminate the unwanted pregnancy for his girlfriend. He tells the provider that they had the casual sex; they had not used any contraception.
- Issues and characteristics to discuss:
  - His parents: very strict and conservative, she does not want them to know anything about this.
  - His girlfriend: 20 years old and is studying bachelor, have been engaged for a year and having occasional physical contacts
  - Sexual history: only had one sexual partner (both)
  - Contraceptive knowledge: thinks that except condom, other methods are not suitable for unmarried boy and girl.
  - Why did she choose to come to MSI? His friend told him that pill to abort MA can be obtained from MSC

Scenario 2

- Personal characteristics: Late 20s, married, but husband works abroad, has 5-year-old child. Open, free thinker, SLC pass (+2 drop out), open minded personality
- Reason for visit: coming to center with amenorrhea for 6 weeks (non-judgmental attitude of SPs, SA and PAFP counseling)
- Issues and characteristics to discuss:
  - Her husband: Supportive of family planning, was using Depo but her husband asked to discontinue before leaving for Abu Dhavi
  - Contraceptive practice: She has used the injection before.
  - Contraceptive knowledge: Has limited knowledge of other FP methods.
  - Why did she choose to come to MSI? Had heard about MSC from her neighbor
  - Onset of menarche: 11/12 years
  - Menstrual history: regular
  - Children: 1 Son (5 years)
  - Obstetric history: had no problems at the time of childbirth. Her son was born in hospital
  - Abortion history: none

Scenario 3

- Personal characteristics: Mid-20s married, has 3-year-old child. Nervous about family planning options, less open-minded personality
- Reason for visit: coming to the centre with husband; has had amenorrhea for 8 weeks.
- Issues and characteristics to discuss:
  - Contraceptive practice: She doesn’t use contraception because she and her husband don’t believe in taking pills. Have heard of a lot of negative side effects of the methods.
  - Contraceptive knowledge: Has some knowledge of a few methods (copper T, pills, condoms)
  - Why did she choose to come to MSI? Had heard about MSC from her neighbor
  - Menstrual history: regular
  - Children: 1 daughter (3 years)
  - Obstetric history: had no problems at the time of childbirth.
  - Abortion history: one

**Questionnaire**

- Basic information:
  - Centre code:
  - Name of centre:
  - Address:
  - Date:
  - Mystery Client ID:
- Scenario enacted:
  - Safe abortion
  - Family planning
  - Age:
  - Parity:
  - Marital status:
  - Other:
- Time at facility:
  - Arrival time:
  - Departure time:
- Did the provider use a series of laminated cards to ask you questions? If so, which questions did the provider ask? (Check all that apply)
  - Do you have any medical issues we should be aware of?
  - Have you used family planning before? Which methods? What did you like or dislike about them?
  - Is your partner receptive to your use of family planning?
  - Would you like to have a child in the future? If so, when?
- Which methods did the provider discuss with you? (Check all that apply)
  - Condoms
  - Oral contraceptive pills
  - Depo Provera shot
  - Injectable, long-action contraceptive
  - Copper IUD
  - Tubal ligation
- Did the provider answer your questions about the methods?
- Did the provider recommend any specific method(s) for you? Which one(s)? What reason did they give for suggesting these methods?
- How many minutes did you spend with the counsellor talking about family planning from start to finish?
- Which of the following words would you use to describe the provider’s disposition (select one from each pair)
  - Warm v Cold
  - Patient v Impatient
  - Calm v Stressed
  - Busy v Bored
  - Friendly v Stern
  - Talkative v Short
- Did the provider do anything else that was notable? Anything that was different from other providers you’ve visited? (Describe below)

**Exhibit S5: Client Exit Interview (CEI) questionnaire**

| **CEI** | **Question Asked** |
| --- | --- |
| % informed about potential side effects | \| **Did the provider tell you about the potential side effects of the service you received?** \| Yes ....................................1  No .....................................0  Don’t know….………..........9 \| \| --- \| --- \| |
| % received follow up instructions | \| **Were you given clear instructions about what to do if you had any problems or side effects as a result of the service received today?** \| Yes ....................................1  No ......................................0  Don’t know….……..............9 \| \| --- \| --- \| |
| % that were satisfied with overall services (good & very good) | \| **How would you rate your overall experience at the facility today?** \| 1                 2              3              4                5  (very poor)    <------------------🡪  (very good) \| \| --- \| --- \| |
| % that would recommend to a friend (likely & very likely) | \| **Based on your experience today, how likely is it that you would recommend this MSI facility to a friend?**  READ EACH OPTION ALOUD. \| Very likely .....................1  Likely .............................2  Neither……………………3  Unlikely……….………….4  Very unlikely…………….5  Not applicable…………..9 \| \| --- \| --- \| |
| % of client that wanted a specific method who got the method of choice | A combination of :   \| When you came here today, was there a specific FP method you wanted to get from the provider?  *(*PROBE*: Before your consultation, did you have a specific method in mind?)* \| Yes ……………………...1  No..................................0  Don’t know...……….......9 \| \| \| --- \| --- \| --- \| \| Which methods did you want to get when you came here today?    ONE ANSWER ONLY. CIRCLE MAIN METHOD, IF MORE THAN ONE IS NAMED. \| A. Female sterilisation (WAFSTER)………  B. Male sterilisation (WAMSTER)…………  C. IUD (WAIUD)……………………….......  D. Implants (WAIMP)………………............  E. Injectables (WAINJ)...............................  F. Contraceptive pills (WAOCP)…………..  G. Male condoms (WAMC)........................  H. Female condoms (WAFC).....................  I. Emergency contraception (WAEC)........  J. Other modern method (diaphragm, foam tablets, spermicidal jelly, vaginal ring, contraceptive patches) (WAOMM).....................................................  K. Breastfeeding  (WALAM)……….………  L. Fertility awareness methods (withdrawal, rhythm, abstinence)(WATFM)…………… \| \| \|  \|  \|  \|   And the PAFP method they received. (The data provided in only for Surgical Abortion clients) |
